# Oral health knowledge and dental behavior among individuals with autism in Jordan: a case–control study

**DOI:** 10.1186/s12903-021-01423-4

**Published:** 2021-02-11

**Authors:** Sabha Mahmoud Alshatrat, Isra Abdelkarim Al-Bakri, Wael Mousa Al-Omari, Noor Abdullah Al Mortadi

**Affiliations:** 1 Department of Applied Dental Sciences, College of Applied Medical Sciences, Jordan University of Science and Technology, Irbid, Jordan; 2 Department of Prosthodontics, Faculty of Dentistry, University of Science and Technology, Irbid, Jordan; 3 Department of Restorative and Prosthetic Dental Sciences, College of Dentistry, King Saud Bin Abdulaziz University for Health Sciences, Riyadh, Saudi Arabia

**Keywords:** Oral care, Dental behavior, Oral health knowledge, Autism spectrum disorder (ASD)

## Abstract

**Background:**

Oral care is acknowledged as an integral component of general health and plays an essential role in establishing the desired level of quality of life for individuals with autism spectrum disorder (ASD).

**Purpose:**

To investigate oral health knowledge and dental behaviors in individuals with ASD in comparison with individuals without ASD in Jordan.

**Methods:**

A case–control study was carried out among 296 caregivers of individuals with ASD (n = 147) and control (n = 149) groups. A closed ended, validated self-designed questionnaire was distributed. The questionnaire included questions addressing participant’s oral health knowledge and behaviour. Data were analyzed using SPSS® software Version 22 with a 0.05 level of significance. A Chi-square test and contingency-table analysis were performed.

**Results:**

Individuals with ASD in Jordan were significantly less knowledgeable about different oral health aspects than individuals without ASD (*p* < 0.05). Fewer individuals in the ASD group brushed their teeth once or twice daily (89%), compared to the control group (93%). Only 15% of the ASD participants could brush their teeth without help. The use of fluoridated toothpaste and the frequency of using mouth rinse demonstrated a significant difference between groups (*p* < 0.05).

**Conclusion:**

ASD individuals in Jordan suffer from a significant lack of oral knowledge comparing to their controls, leading to a misunderstanding of the basic and highly important dental health aspects. Indicating that the knowledge was not enough to influence their dental behaviors. Children with ASD and their families should receive appropriate education in special need oral health care given by oral health professionals to reduce the risk of having dental problems and oral disease and enhance their quality of life.

## Introduction

Autism disorder is a complex developmental disability characterized by persistent neurodevelopmental conditions with early childhood onset [1]. It is characterized by impaired social interaction and communication as well as limited, repetitive patterns of behavior, interests, or activities and unusual sensory interests or sensitivities. Autism disorder has many subtypes that were merged under one definition of Autism Spectrum Disorder (ASD) in 2013 in the Diagnostic and Statistical Manual of Mental Disorders [2]. Individuals with autism vary widely in abilities, intelligence, and behaviors. A wide spectrum of medical and behavioral symptoms is exhibited by children with autism, which makes routine dental care a very challenging task. The unpredictable body movements, hyperactivity, quick frustration, and self-injurious behavior may complicate oral healthcare for autistic individuals [3]. Previous studies reported that sensory sensitivities in children with autism have shown to be related to behavior difficulties in a dental clinic [4]. Bad oral habits, such as bruxism, biting the lips, and tongue thrust, are oral health problems associated with autistic individuals. Moreover, dental caries, delayed tooth eruption, mouth trauma, and injury are common to individuals with ASD [3].

Children with ASD exhibited poorer oral health conditions and higher caries prevalence compared to healthy children [5, 6]. Poor dietary habits, the effects of prescribed drugs taken by individuals with ASD, poor oral hygiene due to difficulty of home care measure for many children or caregivers, and increased risk of erosion due to vomiting as reported by many caregivers are factors that may contribute to poor oral health and high caries level in children with ASD [1, 3, 7].

On the other hand, other studies reported similar caries prevalence [8, 9] or lower caries prevalence and severity in children with ASD compared to controls [10, 11]. The less frequent snacking in individuals with autism might contribute to low caries susceptibility compared to controls [1].

Plaque and gingival scores, the prevalence of periodontal disease, the need for professional scaling, and root planning were higher among individuals with ASD compared to healthy controls [12]. Further, gingival recessions and lower saliva flow were reported in individuals with ASD [1].These results might be attributed to several side effects of prescribed medications taken by some individuals with ASD as pharmacological management for mood disorders, attention insufficiency, aggression, anxiety, and sleeplessness, which might affect the salivary flow and increase the risk of gingivitis and bleeding. Besides, less frequent tooth brushing can contribute to poor gingival health in individuals with ASD [1, 3, 7].

According to the World Health Organization, individuals with disabilities have greater unmet needs for oral healthcare than those without disabilities [13]. Dental healthcare has been identified as leading unmet healthcare priority in children with disabilities [14, 15]. For children with autism, dental healthcare remains the most common unmet healthcare needs [5, 14, 16] Data from Brickhouse reported 19% of children with ASD having unmet dental needs in the last 12 months [16], while a study by Lai et al. [14] showed 12% of children with ASD having unmet dental needs in the last 6 months.

The prevalence of ASD is rising globally. According to the World Health organization, about one in 160 children has ASD worldwide [13]. According to the Centers for Disease Control and Prevention CDC [17] one in 59 children has been identified with ASD in the USA. Tracking the prevalence of ASD in Jordan poses a challenge due to little research and information available on the same. It is estimated that in 2018, there were 8,000 cases of ASD in Jordan [18].

Given that individuals with disabilities are at a higher risk for oral diseases due to underlying congenital anomalies and inability to receive the needed care to maintain oral health [15, 19], regular preventive dental care is essential to maintaining optimal oral health. However, to date, there are no studies conducted in Jordan addressing the dental knowledge, dental awareness, and challenges and barriers to oral care faced by individuals with autism.

Therefore, this study aims to assess oral-health knowledge and dental behaviors among individuals with ASD compared to individuals without ASD.

### Materials and methods

A self-designed, closed-end, validated questionnaire was formulated in the Arabic language, the official language in Jordan. The questionnaire underwent content validity testing. The self-designed questionnaire’s content validity was established by an expert panel consisted of two professional colleagues and the average congruency percentage (ACP) was calculated. The ACP score was equal to 92% indicating this questionnaire is valid for application in this study. Test–retest reliability was achieved by administering the questionnaire twice to the same individuals (n = 10). Cronbach’s alpha was used to test the internal reliability of the questionnaire. Cronbach’s alpha coefficient was equal to 0.75, indicating the items have acceptable internal consistency. The questionnaire was pilot tested using 10 volunteer parents/caregivers of individuals with autism. The questionnaire was presented to the parents/caregivers during a parent–teacher conference at the schools/centers. The parents/caregivers were asked to answer the questionnaire and provide feedback on its content, time, clarity, and format. The questionnaire was revised taking into consideration the comments of the parents/caregivers.

The questionnaire consisted of three sections: five items were used to cover demographic information; 12 items were used to cover information about participant’s oral-health knowledge, and seven items were used to assess the participant’s dental behavior. Information regarding the severity of the disability was based on the diagnosis in the patient's record, which was obtained by the principal of the center from different areas in Jordan.

A convenience sample was used to recruit the participants of the study from different centers located in North, South, and Middle areas in Jordan. A gender-matched sample was recruited for this study (± 5). The sample comprised individuals attending the special-care center. A list of special-care centers associated with ASD was obtained from the Ministry of Social Development. Cases were recruited from 13 different centers from different areas in Jordan. The willingness of the special-care center manager to distribute the questionnaire to the participants was the reason for selecting these settings. The centers were asked to circulate the questionnaire to their members. The questionnaire was made available in print to the participants to answer the questions. A cover letter explaining the study and a copy of the questionnaire was sent to the participants and placed by the teacher in the participant's folder to be filled in at home by individuals with ASD if they were able to read and comprehend or by the parent/caregiver to answer the questions on the respondent’s behalf if they could not.

Participation in the study was voluntary, and full confidentiality of the collected data was ensured. The control group was composed of children/adolescents without ASD from the same geographic area of the centers. The control group (children/adolescents) was selected from the same schools where the cases studied were enrolled. The school principals of the identified schools were informed, and seven of them agreed to participate in this study. In the case group, there were adult individuals; thus, the control group was recruited from residents in the same geographical area of the centers if they agreed to participate. The questionnaire was collected after one to three weeks. After three weeks, those who had not responded were sent a reminder note encouraging them to reply. Six weeks after the initial sending, the deadline was set for the receiving of questionnaires. Two hundred questionnaires were sent to all individuals with autism, out of which 147 questionnaires were filled and sent back. On the other hand, 149 filled questionnaires from individuals without ASD were sent back out of 200 that were sent.

The data was anonymized and de-identified prior to analysis. All data was reported in group form. The software package, SPSS Version 22, was used to analyze the data with a 0.05 level of significance. A descriptive analysis of univariate distributions was obtained for each of the 25 questionnaire items. A chi-square test and contingency-table analysis were performed on the data. As not all respondents answered each question, the denominator used to calculate the proportions was the total number of non-missing values.

## Results

The final sample size of the study was N = 147 for ASD and N = 149 for control with a response rate of 74% for both groups. Regarding the severity of disability among individuals with ASD, results revealed that 28% were mild, 44% were moderate, and 28% had a severe disability. The mean age of the overall sample size was 35 years. More descriptive demographic characteristics of the sample have been presented in Table 1.

**Table 1:** Socio-demographic characteristics of participants with ASD and control group

Characteristics		ASD N (%)	Control N (%)
*Gender*			
Male		105 (71.4%)	100 (67.1%)
Female		42 (28.6%)	49 (32.9%)
*Age*			
≤ 18		103 (70%)	97 (65%)
19–40		33 (22.5%)	37 (25%)
> 40		11 (7.5%)	15 (10%)
*Education*			
Elementary		131 (89.7%)	132 (88.6%)
Middle school		9 (6.2%)	17 (11.4%)
High school		4 (2.7%)	0 (0%)
Collage and more		2 (1.4%)	0 (0%)
*Family Income (JD)*			
< 250		74 (50.3%)	2 (1.3%)
250–500		58 (39.5%)	70 (47.0%)
500–1000		14 (9.5%)	64 (43.0%)
> 1000		1 (0.7%)	13 (8.7%)
*Insurance*			
Yes		109 (74.1%)	100 (67.1%)
No		38 (25.9%)	49 (32.9%)

Respecting the response of the participants to oral-health knowledge, a significant lack of knowledge was detected among individuals with ASD compared to the control group in most of the items (p < 0.05). But no significant difference was found regarding the effect of soft drinks (p = 0.54) and teeth brushing (p = 0.82) on oral health. Table 2 illustrates the other 12 items assessing the knowledge level among participants.

**Table 2:** Oral health knowledge among individuals with ASD compared to the control group

Questions	ASD N (%)	Control N (%)	P-value
*Dental plaque is formed by colonizing bacteria*			
Yes	65 (44.5%)	90 (60.4%)	.022*
No	4 (2.7%)	4 (2.7%)	
Don’t Know	77 (52.7%)	55 (36.9%)	
*Dental caries mainly caused by the bacteria*			
Yes	102 (69.9%)	117 (79.1%)	.016*
No	14 (9.6%)	18 (12.2%)	
Don’t Know	30 (20.5%)	13 (8.8%)	
*Having sugars can lead to dental caries*			
Yes	130 (89.0%)	140 (94.0%)	.018*
No	6 (4.1%)	8 (5.4%)	
Don’t Know	10 (6.8%)	1 (0.7%)	
*Having soft drinks affect dental health*			
Yes	130 (88.4%)	141 (94.6%)	.054
No	5 (3.4%)	5 (3.4%)	
Don’t Know	12 (8.2%)	3 (2.0%)	
*Is there any relationship between oral health and overall health?*			
Yes	131 (89.1%)	125 (83.9%)	.012*
No	3 (2.0%)	15 (10.1%)	
Don’t Know	13 (8.8%)	9 (6.0%)	
*It is normal for your gum to bleed during brushing*			
Yes	51 (34.9%)	31 (20.8%)	.006*
No	84 (57.5%)	112 (75.2%)	
Don’t Know	11 (7.5%)	6 (4.0%)	
*It is normal for your gum to be red*			
Yes	71(48.6%)	49 (32.9%)	.014*
No	63 (43.2%)	89 (59.7%)	
Don’t Know	12 (8.2%)	11 (7.4%)	
*It is normal for your gum to be swelling*			
Yes	20 (13.6%)	5 (3.4%)	.001*
No	115 (78.2%)	139 (93.3%)	
Don’t Know	12 (8.2%)	5 (3.4%)	
*Brushing teeth regularly protects your teeth*			
Yes	131 (89.1%)	142 (95.3%)	.082
No	10 (6.8%)	6 (4.0%)	
Don’t Know	6 (4.1%)	1 (0.7%)	
*You need to visit a dentist only when you have a toothache*			
Yes	56 (38.4%)	32 (21.5%)	.000*
No	78 (53.4%)	116 (77.9%)	
Don’t Know	12 (8.2%)	1 (0.7%)	
*You need a hard toothbrush to clean your teeth*			
Yes	29 (19.7%)	7 (4.7%)	.000*
No	102 (69.4%)	136 (91.3%)	
Don’t Know	16 (10.9%)	6 (4.0%)	
*Dental floss is necessary to keep your teeth clean*			
Yes	90 (61.2%)	113 (75.8%)	.023*
No	29 (19.7%)	20 (13.4%)	
Don’t Know	28 (19.0%)	16 (10.7%)	

*Significant result, *p* < .05 \

The results of the participant’s dental behavior indicated that fewer individuals in the ASD group brushed their teeth once or twice daily (89%), compared to the control group (93%), though the difference was not significant, *p* = 0.126. The obvious significant difference was found for the ability to brush without help, where controls showed 100%, whereas only 15% of the autistic participants were capable of brushing their teeth without help. However, the use of fluoridated toothpaste and the frequency of using mouth rinse demonstrated a significant difference between groups (*p* < 0.05), where 38.1% of the individuals with ASD used mouth rinse for once or more a day, but only 26.8% of the control group did the same. Table 3 demonstrates the response of the participants to dental behavior questions.

**Table 3:** Dental behavior among individuals with ASD compared to the control group

Variable	ASD	Control	*P-*Value
*How often do you brush?*			.126
Once or more	131 (89%)	139 (93.3%)	
Occasionally	16 (11.0%)	10 (6.6%)	
*Ability to brush*			.000*
Completely without help	22(15%)	149 (100%)	
Completely with help	125(85%)	0 (0%)	
*How often do you floss your teeth?*			.511
Once or more a day	56 (38.1%)	25 (16.8%)	
Occasionally	118 (80.3%)	124 (83.2%)	
*How often do use mouth-rinse?*			.039*
Once or more a day	56 (38.1%)	40 (26.8%)	
Occasionally	91 (61.9%)	109 (73.2%)	
*Do you use fluoridated toothpaste?*			.000*
Yes	72 (49.3%)	113 (75.8%)	
No	22 (15.1%)	6 (4.0%)	
		30 (20.1%)	
Don't know	52 (35.6%)		
*Frequency of eating sweets*			.065
Once or more a day	131 (89.1%)	141 (94.6%)	
Occasionally	16 (10.9%	8 (5.4%)	
*Frequency of drinking soda/day*			.000*
1–2 cans	120 (81.6%)	147 (98.7%)	
3–4 cans	18 (12.2%)	2 (1.3%)	
None	8 (5.5%)	0 (0%)	

*Significant result, *p* < .05

## Discussion

Due to increased public awareness and improvements in diagnostic tools and changes in referral patterns, the number of diagnosed cases of ASD has tremendously increased over the past 3 decades [20]. Consequently, this disorder with associated complex behavioral and neurodevelopmental disabilities that impair normal brain function has placed a pressing burden on public health services in various countries. Oral care is acknowledged as an integral component of general health and plays an essential role in establishing the desired level of quality of life for the ASD population [21, 22]. However, oral health has been cited as the most unmet healthcare need among children with special healthcare needs in the United States [23].

According to our knowledge, this is the first study that explores the oral health-related issues in the autistic population in Jordan.

The study gender distribution reflects higher prevalence of ASD in males, which has been previously confirmed [20, 24], where males comprised the majority of the current study population (71.4%).

The participants had comparable elementary- and middle-school educational level. This reflects the readiness of educational institutions to accommodate autistic individuals and shows a positive attitude among the parents towards pursuing an appropriate education to their affected children. This educational level is suggestive that autistic subjects in the current study may be considered intellectually suitable. This is concurrent with a previous study [1] on healthy Swedish individuals; although they were found to have higher educational (university/college) level compared to their ASD counterparts, this difference was not found at elementary- and high-school levels. As the severity of autism was associated with intelligence, ranging from moderate to severe, the same can be applied to the severity of expressed symptoms, which can be mild, moderate, or severe. These variations in intellectual abilities should be taken into account when assessing or planning for oral healthcare of autistic individuals. The variation between individuals implies different abilities and behaviors as well as different needs [25]. However, generally, ASD patients require a high degree of patience, commitment, and a thorough understanding of the level of their intellectual ability [1, 22].

Because dental caries and periodontal diseases are widely common multi-factorial diseases, ASD individuals and parent’s knowledge about dental health and causative factors of dental diseases cannot be overemphasized if prevention of these diseases is to be encouraged. ASD individuals’ beliefs, knowledge, and practice about oral health are thus very essential. This study revealed that knowledge of the major causative factor of dental diseases, namely caries, was sorely deficient in the ASD group. It was evident that a significantly larger number of individuals without ASD were aware that dental plaque is formed by colonizing bacteria compared to individuals with ASD. Similarly, significantly more individuals without ASD had associated caries development with bacterial colonization compared to ASD participants. However, about one-fifth of the ASD subjects reported that they did not even know about bacteria as a potential causative factor for caries, which reflects the inadequacy of knowledge in this area. Caries was previously attributed to irregular and improper tooth brushing rather than to the role of bacteria [24].

Luckily the majority of both ASD and control subjects linked the development of carious lesions with the sugar content of food and beverages. It is more conceivable to the general population to associate sugar with caries rather than be knowledgeable of the role of bacteria. Probably, this simplified causative factor of caries is more disseminated to the general population because it is probably more understandable by the majority of people regardless of their educational and intellectual level. This simplified approach should be further encouraged to increase awareness among the general population and ASD individuals, in particular.

The downside effects of soft drinks on dental health have been raised as a common etiological factor of tooth hard tissue destruction even without bacterial involvement [26]. High consumption of these drinks has been cited as a major dental health-related matter, and all attempts shall be directed towards enlightening young children and adolescents about the destructive consequences of these beverages. Vulnerable subpopulations, such as ASD patients, should be specially targeted. In this study, it was surprising that the great proportion of the ASD participants were aware of the effect of soft drinks on dental health as compared to their controls. This reflects the intellectual abilities of ASD patients, which coincide with their educational level. [1]

Despite the systemic physical and behavioral manifestations of autism, which may reduce the interest of ASD patients and their caregivers in focusing on dental and oral health, the majority of ASD individuals perceived the importance of oral health and its intricate relationship with overall health, which significantly surpassed the perception of the healthy control individuals. Although ASD individuals cannot be mistakenly interpreted as intellectually similar to individuals without ASD, this can rather be explained by the difference in age and educational level between the two groups. This knowledge would substantially enhance the awareness of the prime importance of the prevention of oral diseases as a step towards improving the overall general health and quality of life of patients. The oral health remains the most unmet healthcare needs for autistic patients, which is probably due to the preoccupation of parents and health authorities with the urgency of the medical and systemic sequel of the disease, thus neglecting the oral health needs. The beliefs of the ASD patients and their carers in understanding oral health as key for good general health are important for advancing the preventive measures and investing more in the oral health of this group of individuals.

Healthy individuals have shown increased knowledge about the signs associated with gingival diseases, such as gingival bleeding during brushing, reddening of the gingiva, and gingival swelling. A larger number of ASD patients considered the color of the gingiva, bleeding on brushing, and swelling as normal signs. Dental caries seems to be the most important oral health indicator among the participants with ASD. This result is consistent with previous findings [1, 24]. This indicates a deficiency in dental knowledge relevant to gingival and periodontal diseases, which were reflected in the responses of the ASD participants. In addition to the satisfactory knowledge of ASD patients about the cause of dental caries and the importance of oral health, their knowledge about the necessity of regular tooth brushing was comparable to healthy individuals. However, ASD individuals could not realize the importance of soft toothbrush and flossing role in cleaning their teeth. A significantly larger number of individuals without ASD stated that a hard toothbrush was not needed, compared to ASD individuals. A similar pattern of knowledge was reported for the necessity of the use of dental floss in the two groups. This may represent an influential risk factor for caries and periodontal diseases in individuals with autism [11]. The education of ASD and their parents is paramount for improving the oral health of autistic individuals. It has been confirmed that the parents play a major role in assisting their ASD children to practice more effective oral hygiene measures [27]. Therefore, it is crucial to enforce the understanding of the importance of tooth brushing and flossing in oral health and encourage ASD individuals to embrace both measures as a routine daily practice, despite the expected rejection due to ASD patient’s intrinsic aversion to change [28].

Although some ASD individuals can possess some acceptable intellectual abilities, they can be still considered to have inherent aversion to change and oversensitivity to sensory stimuli, and consequently, they can be overly dependent on their parents for basic oral healthcare necessities [29]. It is, also, well established that autistic individuals lack adequate manual dexterity. This is revealed in the results of the current study. Almost 45% of the patients needed assistance while 40% of the autistic participants relied completely on their caregivers in brushing their teeth. This is consistent with previous studies [11, 27, 30, 31]. Their aversion to brushing their teeth could be explained by the poor manual skills, oversensitivity to sensory stimuli such as tastes or smells, and difficulties in social interaction [32], which creates serious difficulties for caregivers in providing the necessary oral hygiene for autistic children. However, the autistic participants brushed their teeth as frequently as their healthy counterparts in this study, corroborating previous findings [27, 30]. This indicates a high degree of commitment and dedication of the parents to their autistic children’s wellbeing. ASD participants in the current study used less fluoridated toothpaste compared to healthy individuals. Similar findings were previously reported [27]. Unexpectedly, a significantly larger number of ASD participants used mouthwash more frequently compared to healthy participants. This could be attributed to the sensitivity of ASD patients to tastes and smells, and thus, autistic individuals may have been inclined to use mouthwash as a potential alternative to tooth brushing rather than bearing the taste of toothpaste and also given that fewer manual skills are needed compared to tooth brushing. Dipping toothbrush in fluoridated mouthwash has been previously suggested as a proposed strategy to overcome the oversensitivity of ASD children to taste and texture of toothpaste [33].

Interestingly, the number of participants without ASD reported consuming 1–2 cans of soft drinks daily was significantly more than that of the ASD group. However, a larger number in ASD group participants consumed 3–4 cans per day. The dietary habits of ASD individuals were found to be inconsistent. This may be explained by the choosy nature of ASD children when it comes to food. The same may apply to beverages. It is believed that individuals with ASD follow characteristic routines and reject changes [34].

Using self-reported, or caregiver-assisted data can be considered one of the most obvious limitations of the current study. Bias in reporting inaccurate answers or faulty answers in response to misunderstood questions may confound the results. It is maybe stigmatic to admit poor dietary habits or unfavorable oral health behaviors as this is viewed as socially undesirable. However, people with ASD may be less prone to social desirability and may, therefore, be inclined to answer questions with honesty. It is worthy to mention that the majority of previous studies relevant to knowledge and oral health status were conducted on children affected with ASD, while the current investigation was conducted on the adult population. Thus, a comparison between the results of the current study and previous studies should consider the variations between the studied populations.

### Recommendations

There is a lack of knowledge among the ASD group of participants, especially regarding the etiological factor of caries and signs and causative factors of gingival and periodontal diseases. Thus, it is strongly recommended to further the education of individuals with ASD to make a high standard of oral health more achievable. Enforcement of positive behavior among such children is also of high necessity. A system of support should be also established to reach ASD people and their families, and those with special needs individuals should be approached differently. Because ASD children are particularly anxious and exhibit negative behaviors [35], a sensory adapted dental environment may prove helpful to encourage the positive behavior of patients and families [36]. However, this area needs more elucidation in the future.

## Data Availability

The datasets generated during and analyzed during the current study are not publicly available due to containing information that could compromise the privacy of research participants, but are available from the corresponding author on reasonable request.
